# What is the mind map of the hospital’s future changes in a developing country like Iran? A qualitative study

**DOI:** 10.1186/s12913-023-09507-3

**Published:** 2023-07-06

**Authors:** Ali Masoud, Reza Dehnavieh, Vahid Yazdi-Feyzabadi, Atousa Poursheikhali, Somayeh Noori Hekmat, Mohammad kazemi, Mina Ghasemi Moghadam

**Affiliations:** 1 Department of Healthcare Management, Policy and Economics, Faculty of Management and Medical Information Sciences, Kerman University of Medical Sciences, Kerman, 7616913555 Iran; 2 Health Foresight and Innovation Research Center, Institute for Futures Studies in Health, Kerman University of Medical Sciences, Kerman, Iran; 3 Health Services Management Research Center, Institute for Futures Studies in Health, Kerman University of Medical Sciences, Kerman, Iran; 4 Department of Health Services Management, School of Health Management and Information Sciences, Iran University of Medical Sciences, Tehran, Iran; 5 Social Determinants of Health Research Center, Institute for Futures Studies in Health, University of Medical Sciences, Kerman, Iran

**Keywords:** Mind map, Future, Changes, Hospital

## Abstract

**Background:**

Hospitals have a vital role in the future of health systems with upcoming structure, resources, and process changes. Identifying the potential aspects of change helps managers proactively approach them, use the opportunities, and avoid threats. This study presents a mind map of future changes in Iranian hospitals to develop a base for further related studies or prepare evidence for interventions and future-related decisions.

**Methods:**

This study is a qualitative-exploratory one, conducted in two phases. In the first phase, in-depth and semi-structured interviews were conducted to identify future hospital changes over 15 years. The interviews were analyzed using the content analysis method and MAXQDA 2018 software and holding two expert panels to develop the mind map using the 2016 Visio software.

**Results:**

In the first phase, 33 interviews led to 144 change patterns. In the second phase, a mind map of changes was drawn according to experts’ opinions with ten categories: structure and role, knowledge management and research, service delivery, health forces, political and legal, economic, demographic and disease, technological, and values and philosophy, and environmental.

**Conclusions:**

Many changes affecting hospitals rooted in the past continue to the future, but the point is the increasing intensity and speed of changes. Healthcare systems need a systematic approach to monitoring the environment to be updated, agile and proactive. These monitoring systems are essential in providing evidence for Macro-level decision-makers.

## Background

Hospitals are one of the main components of the health system and play an essential role in caring for patients and completing other system functions. They support primary health care providers and the training of health care professionals and researchers. Achieving universal health coverage (UHC) and sustainable development goals requires the commitment of all layers of the health system, including hospitals [1].

Driving forces can challenge hospitals in their strategic and clinical processes while increasing the uncertainty of the future [2]. Trends like rising costs, epidemiological transmission, antimicrobial resistance, the incidence of chronic diseases, aging, urbanization, increasing demand, social and cultural changes, changes in economic conditions and the health market, and competition for attraction and retention of talents and new organizational structure models affect the future of hospitals [3–8]. We are encountering emerging health-related technologies in medical equipment, surgical techniques, various drugs, biological products, and information systems [9]. Health literacy has become more highlighted and is an important public health goal for the twenty-first century [10]. Mixed markets are emerging where general and private hospitals compete to attract public funds and respond to needs [11]. In the past years, attention has been paid to health service providers such as hospitals through collaborative networks [12]. The growth of medical tourism and patients traveling to other countries to receive health services has created new forms of health services. It is changing hospitals’ architecture, design, management, and organization [13].

The mentioned cases are examples of hospital changes regarding the future. This institution’s future has many scopes, dispersion, and changes. It will be vital for managers and decision-makers of the health system to identify and monitor the upcoming changes to manage them proactively. In the past years, Iranian hospitals have undergone many changes, including financial and structural reforms, decentralization, changing payment mechanisms, outsourcing, and increasing the participation of the public and private sectors [14]. Along with the changes, the hospitals have faced challenges like increasing costs more than three times in recent years, which have limited access to services [15]. From a structural perspective, reforms, that mostly have happened regarding dealing with upcoming changes needed to be more comprehensive and aligned with each other or other sections of the health system [16]Human resource management also has changed regarding financing models, work processes, inter-sectoral and intra-sectoral collaboration, and performance evaluation standards [17].

This study tries to identify the future drivers of change that affect hospitals via a systemic approach and map the elements as a mind map. Classifying the drivers into main categories helps analyze the consequences more comprehensively and systematically, and visualizing the data in the form of a mind map enhances visualization of the data and transferring critical messages to the audience.

## Methods

### Study setting

In the present study, Iran hospitals were selected for investigation from the public and private sectors. Iran, with a population of about 85 million people in 2021, is one of the most populous countries in the Middle East region [18, 19]. There are 58 universities of medical sciences under the MOHME that provide services with 935 hospitals and 121,941 beds. Iran has 935 hospitals with 121,941 beds,the ratio is 1.54 beds per 1000 people.

#### Study phases

This study is a qualitative deductive one with an exploratory approach trying to identify the future drivers of hospitals in Iran consisting of two main phases. The first phase was collecting qualitative data and determining the coming changes in hospitals, and the second was mapping the elements as a mind map. The stages and their steps are presented as follows.

#### Collecting qualitative data and determining the future changes in hospitals

To collect future changes in hospitals, open and semi-structured interviews were conducted. All the participants were selected using a purposeful and snowball approach in five groups: Ministry of Health and Medical Education (MOHME), health insurance (HI), Health care providers (HP), Hospital Management (HM), and Researchers and members (RFM). Five experts, one per category, were identified to start the snowball method, and interviews continued till saturation. In determining the participants, the main inclusion criterion was to cover the health system sub-systems as much as possible. The following criterion was having more than five years of related work experience or being active in research or education in the health system’s future. The interviews were virtually using Microsoft Team, Sky roam, and phone, and in-person and the interviewer were in the Kerman province of Iran.

At the beginning of this phase, in-depth interviews were conducted with the purposeful sampling approach. The main question of the in-depth interview was: What are the main aspects of hospital changes in the next 15 years? The reason for choosing a 15-year time horizon is that numerous studies have worked on determining the future changes in the health system and hospitals; they chose the time range between 12 and 20 years for their research and pointed out that the horizon is suitable for examining future changes [20–22]. After content analyzing the interviews, the following interviews were semi-structured. All interviews were conducted between April and July 2021. The duration of the interviews varied from 22 to 80 min. At the beginning of all the interviews, the participants were aware of the purpose of the study, and informed consent was obtained from them. The interviewing continues till saturation and the transcription of the recorded interviews was analyzed with content analysis, the approach suggested by Erlingsson and his colleagues [23], and using MAXQDA 2018. All the authors were involved in the development of the interview guide. The peers did audio taping, transcribing and coding.

#### Developing the mind map

A mind map model helps provide efficient, accurate, and creative solutions. This tool can expand thinking in all fields of education, work, and human life [24] by visualizing insights related to the subject of study and developing relationships between ideas. Concepts, keywords, colors, and images can be used to prepare a mind map while the vital concept is placed in the center, and other concepts branch from it [25, 26]. A mind map is rooted in cognitive structures, and understanding this map is easier for the audience than writing texts [27].

The purpose of the second phase was to categorize the ideas and draw a cognitive map of future hospital changes. Two expert panels were held in October 2021, and the second was 11 days apart virtually with the sky room platform. The participants in this phase were selected purposefully among the first phase participants based on the inclusion and exclusion criteria.

The first panel presented the changes resulting from phase one to experts. The experts categorized the changes and labeled them by the facilitator’s guide. With permission, note-taking was done during the meeting by two researchers. After finishing the first panel, the research team drew a mental map of the hospital’s future changes using Visio 2016.

After mapping the mind map, the second meeting of the expert panel was held. The drawn map was presented during the second session, and the participants were issued potential enhancement or edition points. Finally, the amendments were applied according to the expert’s opinion.

## Results

To determine the future changes affecting the hospitals, 33 people were interviewed in the first phase 19 were male, and 14 were female, with an average age of 45. By content analyzing the interviews, 144 elements of change were identified, presented in Table 3. Repetition in subcodes shows that some characteristics of future hospitals are multifaceted and affected by several aspects of changes. The characteristics of the participants in this phase are shown in Table 1.

**Table 1:** Characteristics of participants in phase one

Organization	Role/task	Number
Ministry of Health and Medical Education (MOHME)	Specialists of Research and Technology Vice-Chancellor Specialists of the Vice-Chancellor of Education Specialists of the Vice-Chancellor of Treatment Specialists of the Deputy Food and Drug Administration Specialists of the vice president of development and management and planning	7
health insurance (HI)	Specialists of the Social Security Health Insurance Organization Specialists of Iran Health Insurance Organization	5
Health care Provider (HP)	General practitioner specialist Nurse	5
Hospital Management (HM) (both public and private hospitals)	Hospital managers Hospital supervisors Officials of quality improvement units	7
Researchers and faculty members (RFM)	Researchers in the field of health economics, health service management, health technology assessment, and futures studies in health	9

In the second phase, 12 experts were selected among the participants of the first phase. Table 2 shows the participants’ characteristics in the panel of experts presented in Table 2.

**Table 2:** Details of participants in the expert panel

Organization	Role/Tas	Number
Ministry of Health and Medical Education (MOHME)	Vice President of Development and Management and Planning	1
health insurance (HI)	expert of Iran Health Insurance Organization	2
Health care Provider (HP)	General practitioner	1
Hospital Management (HM)	Hospital managers (both public and private hospitals)	2
Researchers and faculty members RFM	Health services management, health economics, health technology assessment, and futures studies in health	7

**Fig. 1:**
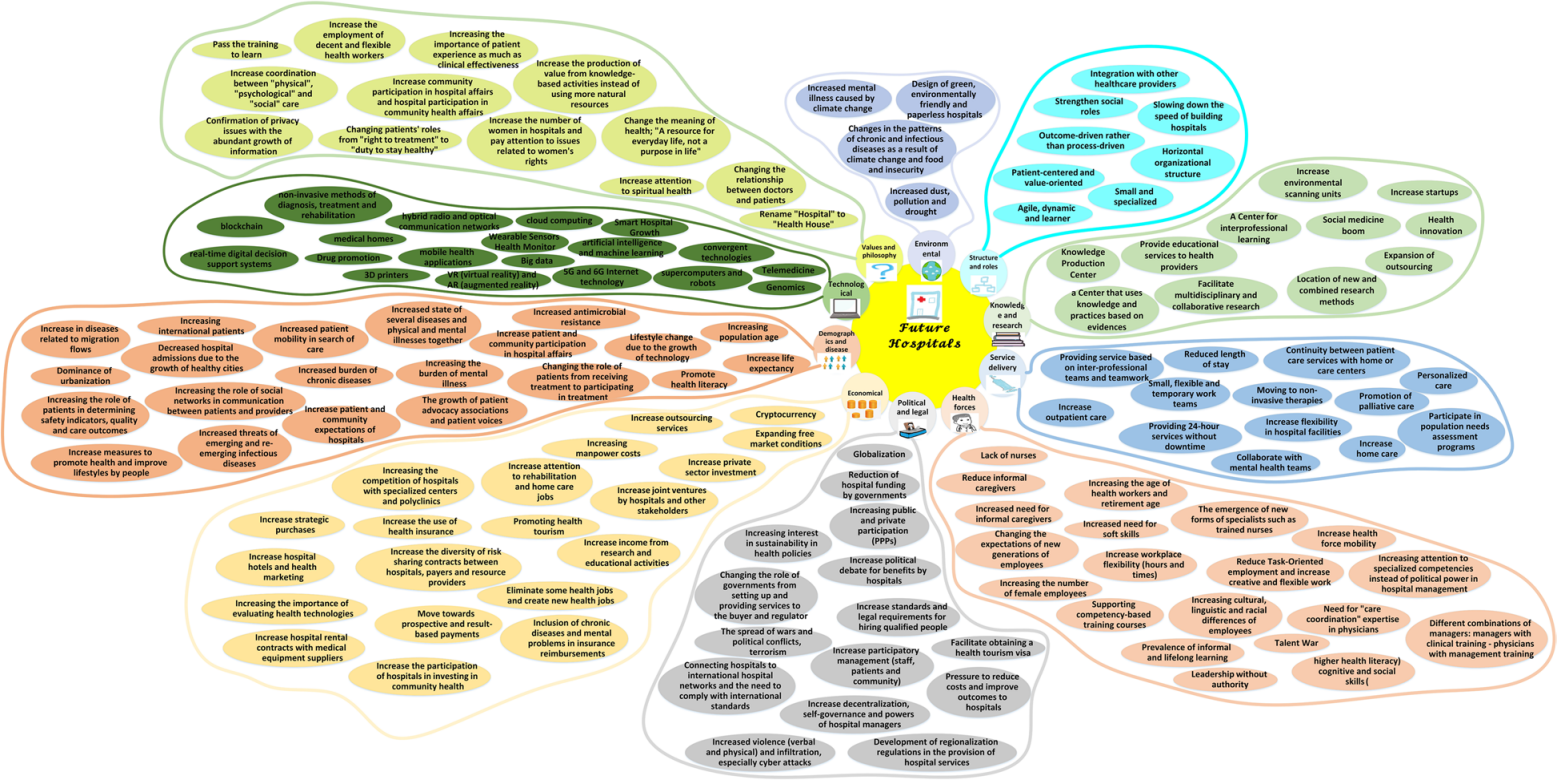
The Mind map of the hospital’s changes in future

To prepare the mind map framework, the experts categorized the future changes into ten main categories of: 1) structure and role; 2) knowledge and research management; 3) service delivery; 4) Health forces; 5) political and legal issues; 6) economic; 7) population and disease; 8) technological issues; 9) values and philosophy; and 10) environmental issues. The explanations of each category and its map is mentioned as follows. The whole map consisting all the categories is presented in Fig. 1 and the codes and themes are presented in Table 3.

**Table 3:** Themes and codes obtained from interviews content analysis

	**Theme**	**Code**	**Quotation examples**
1	Structure and roles	Agile, dynamic and learner	"*In the future, hospitals will have the power to make rapid decisions and take control of their fate. They’ll get rid of complex organizational structures to become more agile and efficient, which will lead to greater independence…"* (M20) *"Hospitals will outsource services that can be provided by others, and they’ll only accept specialized and complicated cases. Their bed sizes and numbers will also decrease*…" (P8) "*Since hospitals play a crucial role in promoting healthy communities, they must collaborate with various stakeholders to achieve this goal. After all, it’s impossible to do it alone…"* (M21)
		Patient-centered and value-oriented	
		Small and specialized	
		Horizontal organizational structure	
		Outcome-driven rather than process-driven	
		Strengthen social roles	
		Slowing down the speed of building hospitals	
		Integration with other healthcare providers	
2	Management of knowledge and research	Knowledge Production Center	
		A Center that uses knowledge and practices based on evidences	"*In the future, providers will work together in collaborative teams and share information and learning with each other*…" (M 36) "*Due to demographic changes and illness, as I mentioned earlier, the root of many of our problems in patient communities and their living environments will be affected. Therefore, there will be a high demand for social medicine*…" (P10) "*As it will be possible to collect and analyze a large volume of expert opinions through technology, this will be a valuable opportunity for problem-solving*…" (HM 9) "*Health systems will provide innovative solutions to address issues and discover new opportunities, creating a platform for start-up activities…*"(P 4)
		Provide educational services to health providers	
		Facilitate multidisciplinary and collaborative research	
		A Center for interprofessional learning	
		Location of new and combined research methods	
		Increase environmental scanning units	
		Social medicine boom	
		Expansion of outsourcing	
		Increase startups	
		Health innovation	
3	Service delivery	Providing service based on inter-professional teams and teamwork	"*As chronic diseases and aging are on the rise, it can be predicted that the need for palliative care will increase, and hospitals are expected to provide these services…"* (RFM 7) *"Technology and diagnostic and treatment methods will advance, and treatment processes will be provided with minimal harm*…" (HI2) *"Patients will become capable of self-care, technology will help monitor their vital signs, and hospitals will have the necessary communication and support so that patients can stay at home…" (RFM3)* "*We may no longer be facing the doctor with its modern concept. We face a health team that shares information and makes decisions together*" (MOHME 4)
		Increase outpatient care	
		Reduced length of stay	
		Small, flexible and temporary work teams	
		Providing 24-h services without downtime	
		Continuity between patient care services with home or care centers	
		Moving to non-invasive therapies	
		Increase flexibility in hospital facilities	
		Collaborate with mental health teams	
		Promotion of palliative care	
		Increase home care	
		Personalized care	
		Participate in population needs assessment programs	
4	Health forces	Lack of nurses	"*I think there is a shortage of nurses now and in the future we will have a problem with the shortage of nurses. In 20 years, when our population ages, how many nurses will we have? Our nursing capacity now is only one-tenth of what we need…*" (HP4) "*The population is aging, so naturally it will lead to an increase in the age of healthcare workers. As the ratio of older people to younger people increases, a mechanism for increasing retirement age will be needed to compensate for the shortage of human resources*…" (RFM1) "*The retirement age will increase to compensate for the shortage of personnel and skills*…" (HI2)
		Reduce informal caregivers	
		Increasing the age of health workers and retirement age	
		Increased need for informal caregivers	
		Increased need for soft skills	
		Changing the expectations of new generations of employees	
		Increasing the number of female employees	
		The emergence of new forms of specialists such as trained nurses	
		Increase workplace flexibility (hours and times)	
		Supporting competency-based training courses	
		Increase health force mobility	
		Reduce Task-Oriented employment and increase creative and flexible work	
		Increasing cultural, linguistic and racial differences of employees	
		Prevalence of informal and lifelong learning	
		Increasing attention to specialized competencies instead of political power in hospital management	
		Need for "care coordination" expertise in physicians	
		Talent War	
		Leadership without authority	
		Different combinations of managers: managers with clinical training—physicians with management training	
		higher health literacy(cognitive and social skills)	
5	Political and legal issues	Globalization	"*Resource constraints and competition will increase the need for political maneuvering by managers to secure benefits." (Source 10)* *"The healthcare system will be pursuing marketing, and they will receive help from the government to facilitate this, including easy visa acquisition for medical purposes as one of the privileges…*" (RFM8) "*Health systems will pay close attention to service grading and regionalization in order to control their services and offer them in a targeted manner*…" (RFM2) "*The hospital independence plan has been underway for several years now, and in my opinion, it will lead to greater decentralization, self-reliance, as well as increased responsibilities and authorities for managers in responding more effectively to local needs in the future*…" (HP5)
		Reduction of hospital funding by governments	
		Increasing public and private participation (PPPs)	
		Increasing interest in sustainability in health policies	
		Increase political debate for benefits by hospitals	
		Changing the role of governments from setting up and providing services to the buyer and regulator	
		Increase standards and legal requirements for hiring qualified people	
		The spread of wars and political conflicts, terrorism	
		Increase participatory management (staff, patients and community)	
		Facilitate obtaining a health tourism visa	
		Pressure to reduce costs and improve outcomes to hospitals	
		Increase decentralization, self-governance and powers of hospital managers	
		Development of regionalization regulations in the provision of hospital services	
		Connecting hospitals to international hospital networks and the need to comply with international standards	
		Increased violence (verbal and physical) and infiltration, especially cyber attacks	
6	Economic	Cryptocurrency	"*The future of money belongs to digital currencies. Currently, mining these currencies requires a lot of energy, but in the future, I believe there will be alternatives for other common currencies*…" (HI3) "*In the future, command economies and government intervention in the economy will be reduced as much as possible for better health*…" (HM6) "*Along with the elimination of some healthcare jobs, others will thrive, such as home care and rehabilitation services. Especially since families will no longer be as densely populated as before and individuals will need assistance*…" (HI5) "*Our resources are limited, so we need to make our purchases more targeted in the future. In my opinion, the field of health technology assessment will become more important in the future*…" (RFM1)
		Expanding free market conditions	
		Increase outsourcing services	
		Increase private sector investment	
		Increasing manpower costs	
		Increase joint ventures by hospitals and other stakeholders	
		Increase income from research and educational activities	
		Increasing the competition of hospitals with specialized centers and polyclinics	
		Increase attention to rehabilitation and home care jobs	
		Promoting health tourism	
		Inclusion of chronic diseases and mental problems in insurance reimbursements	
		Eliminate some health jobs and create new health jobs	
		Increase strategic purchases	
		Increase the use of health insurance	
		Increase the participation of hospitals in investing in community health	
		Increase the diversity of risk sharing contracts between hospitals, payers and resource providers	
		Increase hospital hotels and health marketing	
		Increase hospital rental contracts with medical equipment suppliers	
		Move towards prospective and result-based payments	
		Increasing the importance of evaluating health technologies	
7	Population and disease	Increasing population age	"*The population, their age, gender, and race are constantly changing. Now, issues related to transgender individuals have also been raised. Well, these will definitely have an impact on the healthcare system*…" (HP2) "*Nowadays, many of our patients are well-educated and informed about their rights. They have gathered information from various sources, even if it is limited, about their illnesses. They comply with treatment instructions better than before, and in my opinion, this is a positive move towards better health for the population. However, there are also issues that we face, such as patients who cannot tolerate long waiting times like before. They are sensitive to quality and safety. One important point that they emphasize is that their respect should be fully respected*…" (HM4) "*In recent years, lifestyle changes have begun, and I believe these changes will become more severe in the future. Chronic and mental illnesses will increase due to these changes…*" (RFM1) "*The change in health preferences, such as people’s interest in beauty and fitness services, will increase*…" (RFM9)
		Increase life expectancy	
		Lifestyle change due to the growth of technology	
		Increasing the burden of mental illness	
		Promote health literacy	
		Increased antimicrobial resistance	
		Increase patient and community participation in hospital affairs	
		Changing the role of patients from receiving treatment to participating in treatment	
		Increased state of several diseases and physical and mental illnesses together	
		The growth of patient advocacy associations and patient voices	
		Increased burden of chronic diseases	
		Increasing international patients	
		Increased patient mobility in search of care	
		Increase patient and community expectations of hospitals	
		Increase in diseases related to migration flows	
		Decreased hospital admissions due to the growth of healthy cities	
		Increasing the role of social networks in communication between patients and providers	
		Increased threats of emerging and re-emerging infectious diseases	
		Dominance of urbanization	
		Increasing the role of patients in determining safety indicators, quality and care outcomes	
		Increase measures to promote health and improve lifestyles by people	
8	Technological issues	Telemedicine	"*There is another serious mega trend, and that is our world becoming increasingly dependent on technology as we move forward. This dependence, which has been growing since the communications and technology revolution, will continue to be discussed in more detail in the future. All aspects of our lives are affected by this technological field, and perhaps in the not-too-distant future, the healthcare sector will also become more dependent on it*…" (RFM3) "*I believe the use of cryptocurrencies and technologies such as blockchain in financial and informational transfers in hospitals will increase. Consider that with the growth of big data and its increasing use, blockchain can be used to preserve this information in the healthcare system…" (HP4)* *"The discussion of stem cells, I can confidently say that very powerful databases have been prepared in this field worldwide*…" (MOHME2) "*Technology convergence—cognitive, biological, informational, and nanotechnology—is expected to bring about very serious changes and innovations in the future*…" (RFM6) "*3D printers have now entered the market, and in my opinion, in the future these printers will revolutionize the production of transplant organs for patients. Stem cells also play a helpful role here*…" (HM1)
		Genomics	
		Convergent technologies	
		Supercomputers and robots	
		Smart Hospital Growth	
		Artificial intelligence and machine learning	
		5G and 6G Internet technology	
		Cloud computing	
		Big data	
		VR (virtual reality) and AR (augmented reality)	
		Wearable Sensors Health Monitor	
		Hybrid radio and optical communication networks	
		Mobile health applications	
		Non-invasive methods of diagnosis, treatment and rehabilitation	
		Medical homes	
		Drug promotion	
		3D printers	
		Block chain	
		Real-time digital decision support systems	
9	Values and Philosophy	Rename "Hospital" to "Health House"	"*Hospitals around the world are moving towards becoming not just places for treating illnesses, but also places where people go to improve their overall health, prevent future diseases, or receive services to help them recover*…" (RFM2) "*Patients and doctors will have a two-way interaction and relationship with each other to solve health problems, and patients will no longer be seen solely as customers or recipients of service*…" (HP3) "*Universities and formal education will become less prominent. Nowadays, people voluntarily identify their own needs and take online courses. Platforms such as Coursera and YouTube offer learning content…"* (HM5)
		Changing the relationship between doctors and patients	
		Change the meaning of health; "A resource for everyday life, not a purpose in life"	
		Increase attention to spiritual health	
		Increase the production of value from knowledge-based activities instead of using more natural resources	
		Increase the number of women in hospitals and pay attention to issues related to women’s rights	
		Increasing the importance of patient experience as much as clinical effectiveness	
		Increase community participation in hospital affairs and hospital participation in community health affairs	
		Changing patients’ roles from "right to treatment" to "duty to stay healthy"	
		Increase the employment of decent and flexible health workers	
		Confirmation of privacy issues with the abundant growth of information	
		Pass the training to learn	
		Increase coordination between "physical", "psychological" and "social" care	
10	Environmental issues	Increased dust, pollution and drought	"*One of the other trends that can be significant is the discussion of environment, which is happening due to the changes in the environmental and climatic conditions, leading to changes in the type of ecological diseases. Well, the type of disease that has changed will lead to changes in health-related professions and healthcare management will also require special conditions*…"(HP4) "*Climate change, drought, global warming, and even food and water insecurity that we are currently witnessing have the potential to change patterns of chronic and infectious diseases…"* (RFM7) "*Now the world has moved towards using renewable energy to supply hospitals with energy. They produce the least amount of waste, use natural light for building lighting, and do not use items such as paper…"* (HP2)
		Changes in the patterns of chronic and infectious diseases as a result of climate change and food and insecurity	
		Design of green, environmentally friendly and paperless hospitals	
		Increased mental illness caused by climate change	

### Structure and roles

**Fig. 2:**
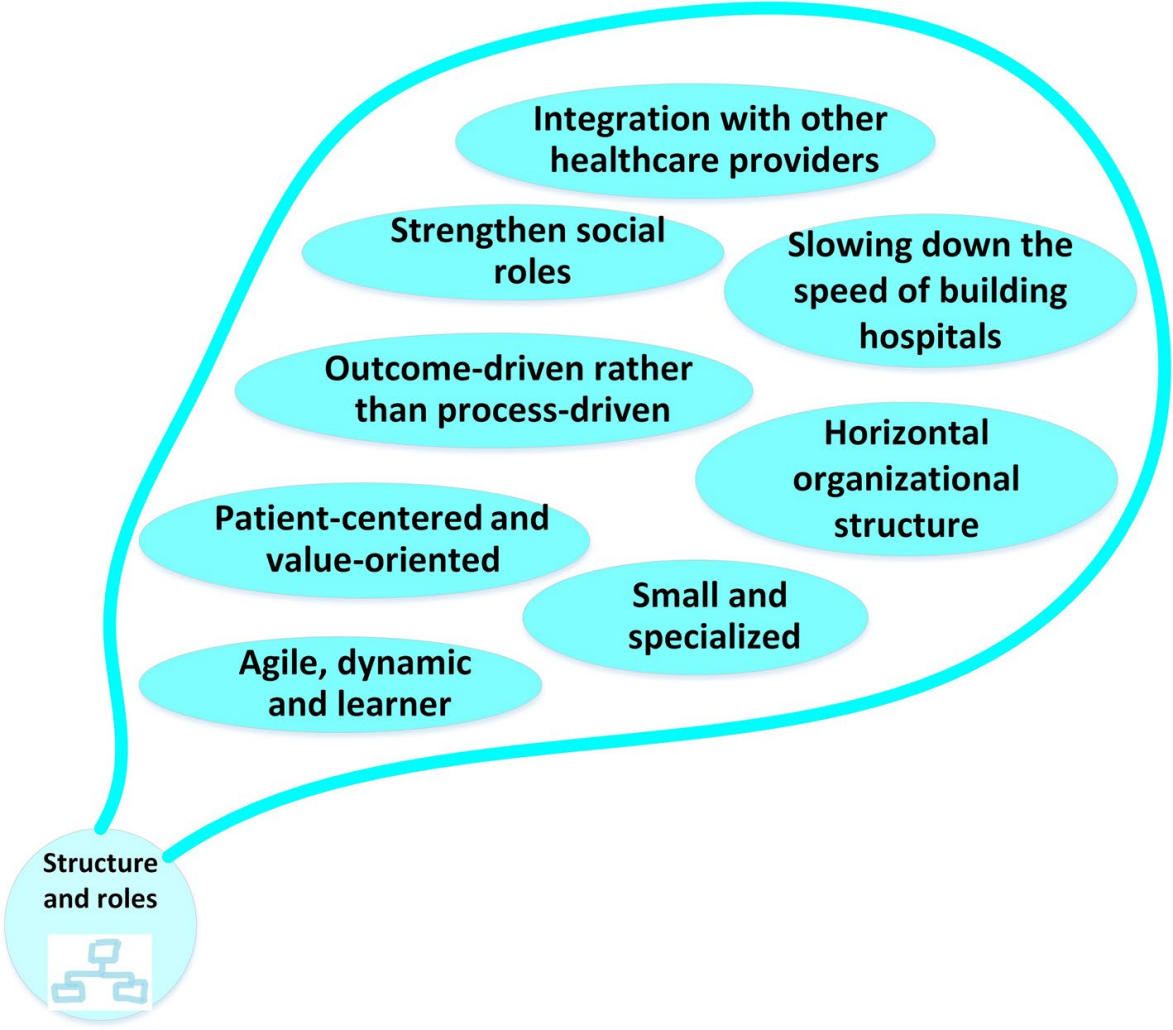
Changes in structure and roles

This code defines the changes that modify the structure of the future hospitals and their roles regarding different stakeholders like society. The related codes are presented in Fig. 2. Participants stated that hospitals would become smaller but more specialized in the future. "…*hospitals will only focus on patients for whom the other health system providers will not have the necessary facilities and expertise, and patients with complex conditions will be referred to them from other levels, and simpler cases will also be at other levels. To be treated*…" (MoH).

Hospitals will have an active, patient-oriented, and user-friendly social role." *Providing services will not be limited to hospitals. Hospital teams in the community will actively promote the lifestyle and treatment of patients in their homes…” (HP). "Due to factors such as the growth of technology and information; The quality and safety of services will increase, the preferences of patients will be taken into account in the design of spaces, processes, and services of hospitals*…" (HM).

### Knowledge and research

**Fig. 3:**
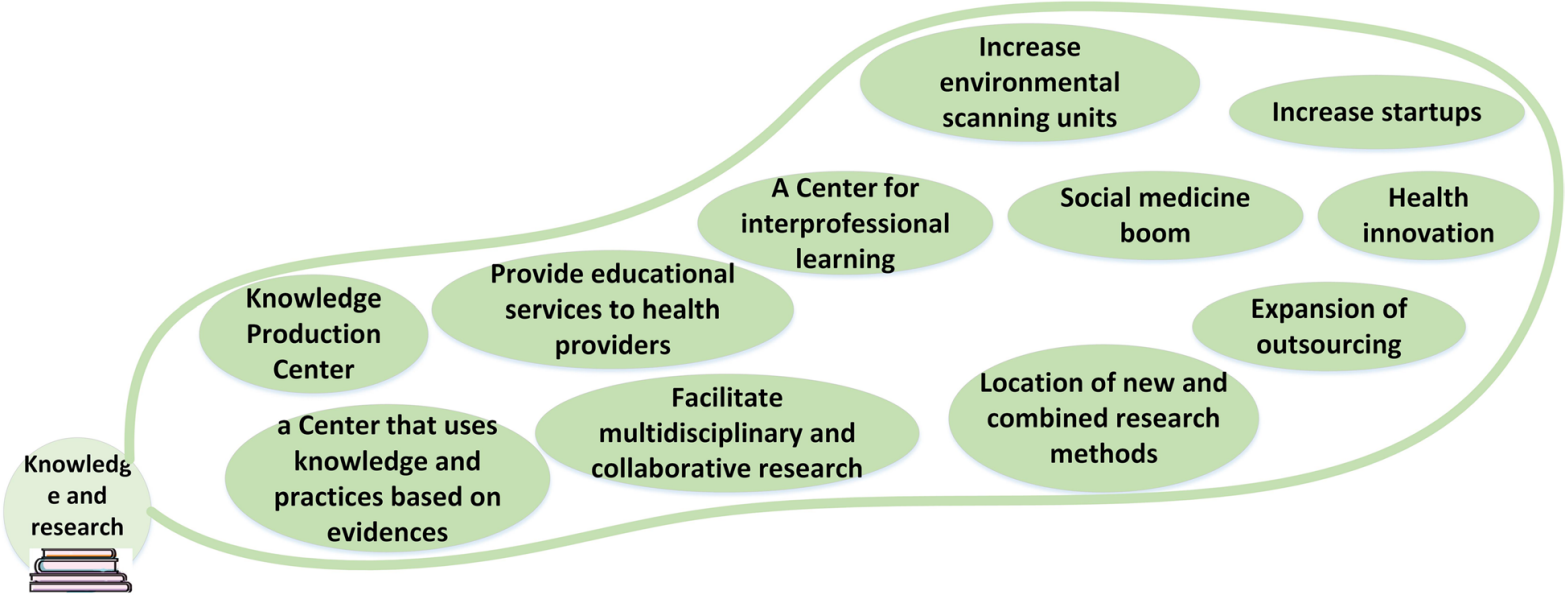
Changes in knowledge and research

This category examines the evolving roles and position of future hospitals in the realm of knowledge management and research. As knowledge continues to rapidly advance, future hospitals must adapt and restructure accordingly. In order to remain relevant, they will need to transform into knowledge-producing centers that prioritize interprofessional learning. To achieve this, hospitals will need to activate environmental scanning units, which will enable them to stay abreast of new developments and emerging trends. The items related to changes in knowledge and research are shown in Fig. 3. *"Hospital administrators will be aware of the importance of political, social, and economic changes in the hospital environment and will monitor them. I believe future hospitals will establish active environmental monitoring units" (HM).*

### Service delivery

**Fig. 4:**
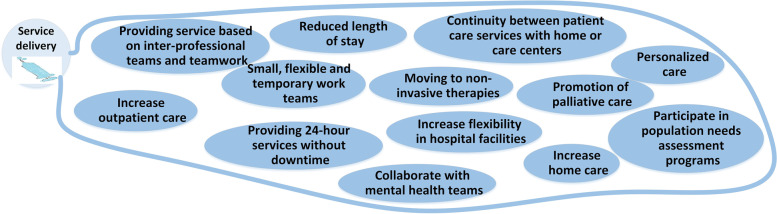
Service delivery changes

This category shows the changes in providing services in future hospitals. The map of the service delivery section is presented in Fig. 4. The future services of hospitals will focus more on home care, palliative care, personalized care, and reduced length of stay. "Th future hospitals will be smaller, the length of stay will be shorter, and they will only accept patients with complex conditions, and the rest will be sent to providers of other levels" (M 35).

### Health forces

**Fig. 5:**
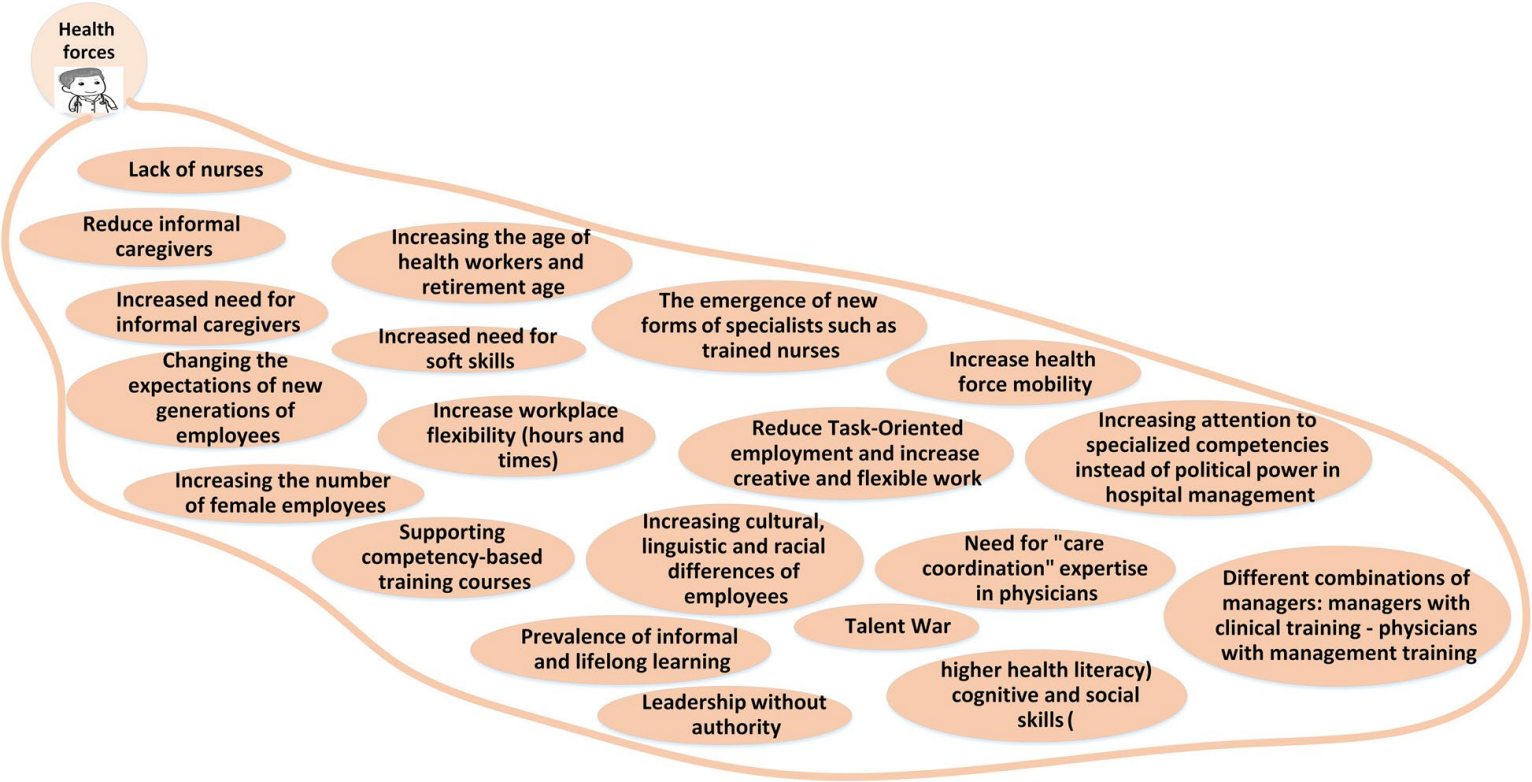
Health forces changes

This theme refers to the changes that will happen regarding the health workforce in the future and the way that that affects the future of hospitals. The health forces will change in the number and nature of their work processes. There will be an increase in the patient’s and health force’s mobility. "*Due to megatrends such as globalization or trends of increasing immigration and urbanization, we will see patients and health workers with different cultures from different cities and countries every day*…" (RFM). Also, A modification in the characteristics of the health workforce will occur. "*With the arrival of the millennial generation, we will see rapid and large changes in employees’ expectations from work environments. The retirement age will increase to compensate for the lack of workforce and skills*" (MoH). Health forces codes are presented in Fig 5.

### Political and legal

**Fig. 6:**
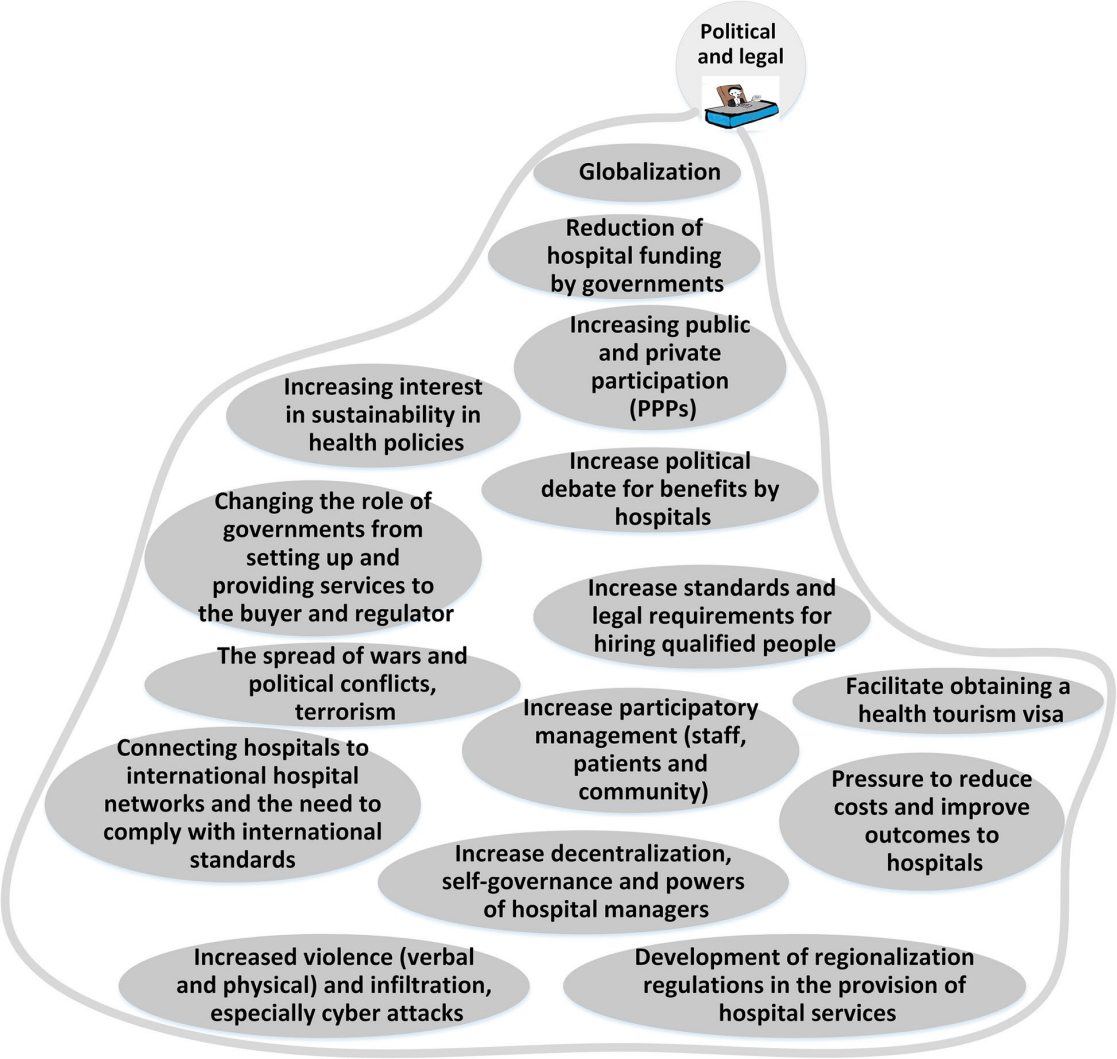
Political and legal changes

The political and legal elements of change are presented in Fig. 6. Changes in developed countries will happen faster than in developing countries. Also, the rate of change has increased compared to the past*." In my opinion, due to trends such as globalization and international communication, several changes are common between developed and developing countries, such as technologies; the only difference is that usually, these changes in those [developed] countries have either already happened or their speed and* intensity *are more significant than in developing countries…, another point that comes to my mind is the speed of changes. We constantly change in the workforce. Or we had the expectations of the patients, but the only thing that has happened now is that due to the growth of technologies such as the media and the Internet, their speed has now increased*…" (RFM). Another point that can be extracted from interviews is that some changes will facilitate and accelerate other changes. For example, developing health monitoring technologies will enable the development of home care services for patients.

### Economic

**Fig. 7:**
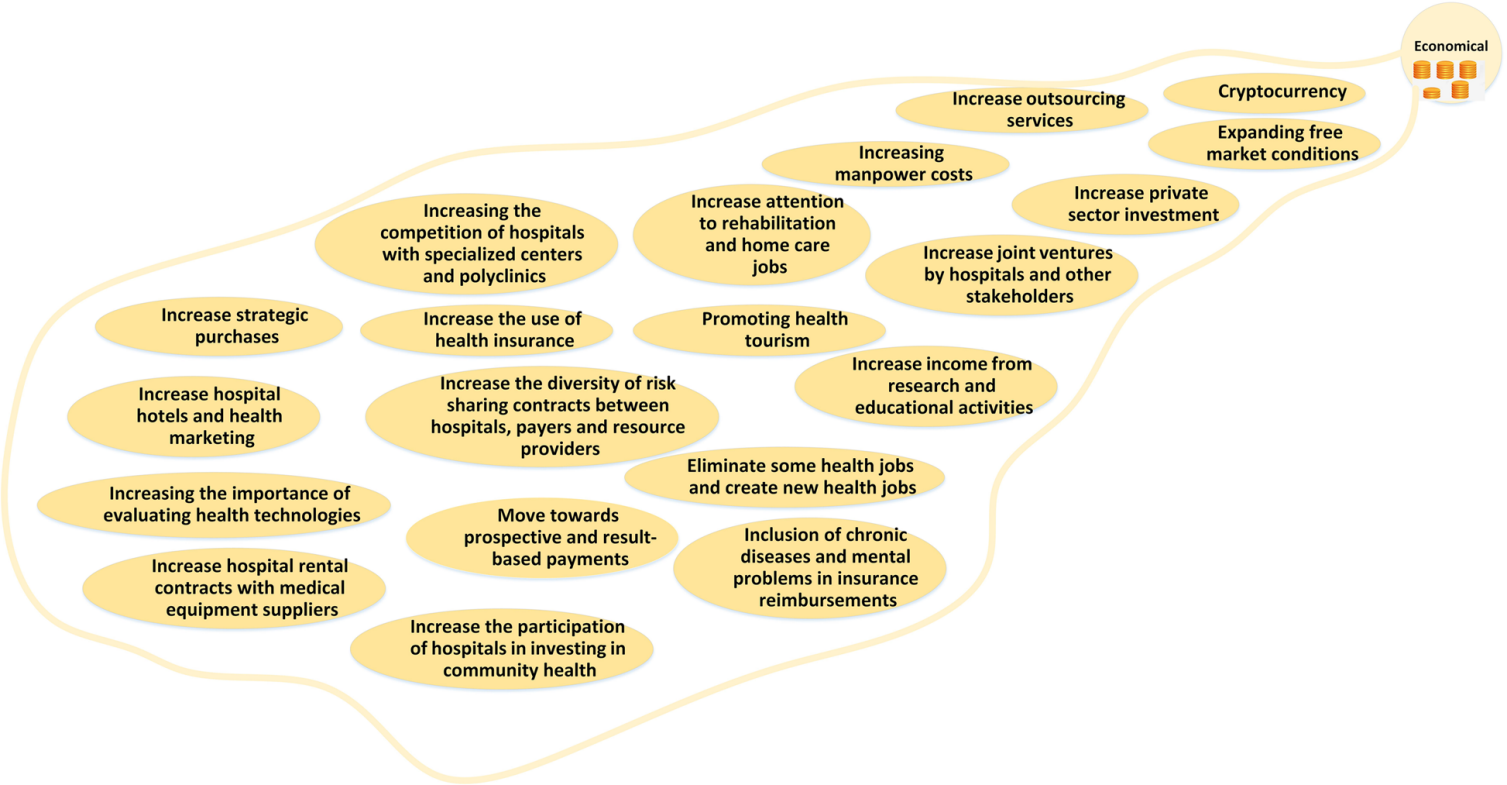
Economical changes

The codes of the economic theme are presented in Fig. 7, like changes in public and private role. The role of the private sector and attention to financial efficiency will increase due to the lack of resources and the increase in technological costs*. "Hospitals will increase partnerships with private sectors and non-governmental organizations to attract more resources…, strategic purchasing will be done more carefully" (HI).*

### Population and disease

**Fig. 8:**
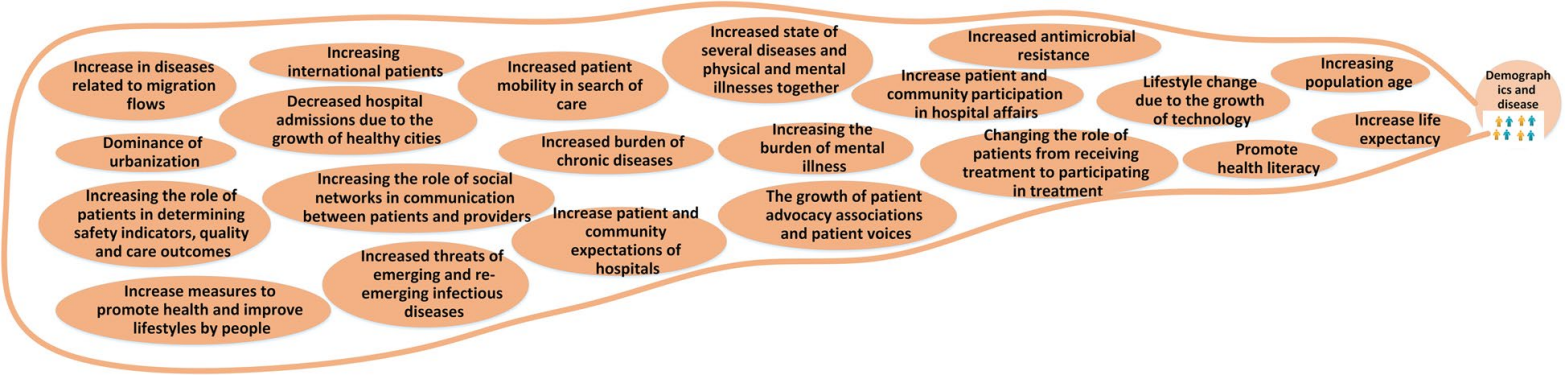
Demographic and diseases changes

The demographic and diseases-related elements are presented in Fig. 8, like changes in the composition and pattern of diseases*. "Clearly, due to the increase in life expectancy and the population aging, it can be predicted that the burden of chronic diseases will increase, even in my opinion, the state of multiple diseases and physical and mental diseases will increase together…" (RFM). "Climate changes, drought or global warming and even water and food insecurity that we are witnessing now, can potentially change the patterns of chronic and infectious diseases" (RFM). "The experience of Covid-19 also showed that the threats of emerging and re-emerging infectious diseases can always exist, so future hospitals should not neglect these issues" (MoH).*

### Technology

**Fig. 9:**
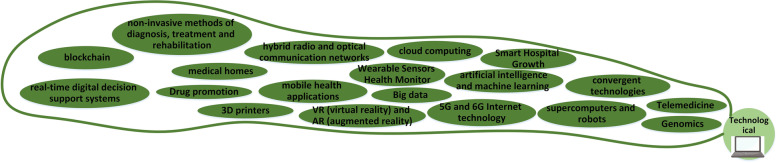
Technological changes

The technological-related elements of the map are presented in Fig. 9. The growth of technology will move forward faster and affect all sectors of the healthcare system*. "In my opinion, using cryptocurrencies and technologies such as blockchain will increase hospitals’ financial and information transfers. It would help if you considered that with the growth of big data and the increase in its use, blockchain could be used to maintain this information in the health system" (HI).*

"In the past years, we consider a significant growth in health technologies, wearable health sensors that help patients control health parameters are one of them. I hope hospital information systems will use this information in the future" (HP). "The development of information analysis methods such as cloud computing can greatly help health systems analyze big data" (RFM).

### Values and Philosophy

**Fig. 10:**
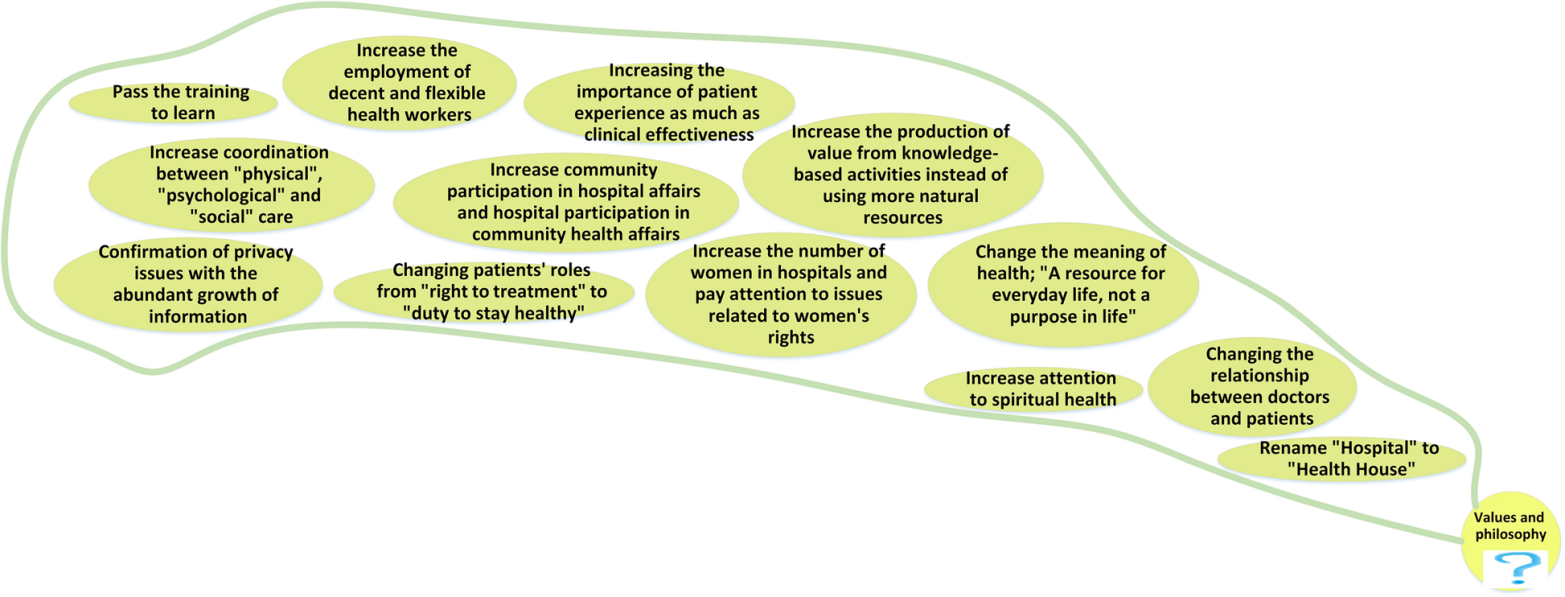
Values and philosophy

The values and philosophy-related elements are presented in Fig. 10, consisting of change in norms and values. *"Associations supporting patients’ rights will become more and more active. Due to the growth of social networks, patients will be able to form a network with each other and express their expectations loudly" (RFM). "The problems of life and the rush and stress that people have now will cause them mental issues; in my opinion, attention to spiritual health will also increase" (HP).*

### Environment

**Fig. 11:**
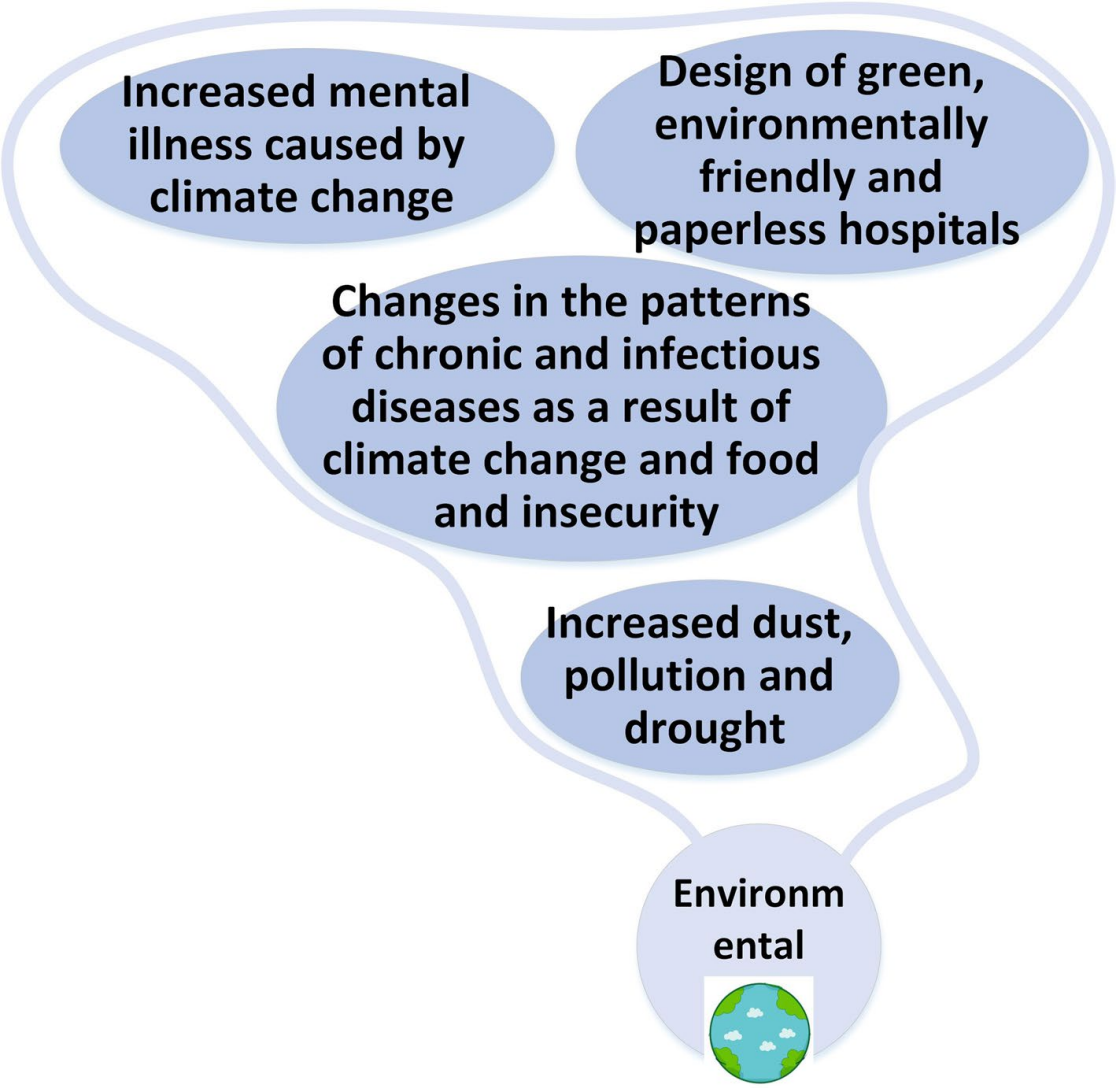
Environmental changes

Green hospitals will be considered and on the other side the crisis like drought, climate change and pollution will increase the burden of diseases like mental health related ones. More information of the environmental future changes are presented in Fig. 11. "*Climate changes, drought and global warming and even food and water insecurity that we are witnessing now have the ability to potentially change the patterns of chronic and infectious diseases*" (M 24).

## Discussion

The speed of changes in the twenty-first century is faster than in previous years [28]. Healthcare is not excluded from these changes and is one of the most complex and growing industries worldwide [29]. Hospitals are also one of the critical components of health systems. Hospital services constitute about 50% of health costs [11]. It is necessary to pay attention to hospitals to improve the overall performance of the health system [30]. The present study showed that the future of hospitals is affected by many factors interacting inside and outside the hospital’s scope and health system.

### Structure and role

In the future, hospitals will become more specialized and smaller where services will be provided merely by their service providers. Less technical and palliative services will be transferred to primary care providers, nursing centers, or patients’ homes. In the health system profile of Iran, developed by WHO, home-care services are emphasized due to aging and increasing chronic diseases [31]. Besides, integrating specialized services in hospitals will keep them in the service delivery line to meet society’s needs. Their number will be reduced, or they will not be constructed without a needs assessment. Barber also points out in his study that establishing hospitals will be slower in the future, and they will probably face stricter controls on construction and size [32]. Also, the congestion in hospitals will decrease over time. However, diseases and more complex treatment methods will remain in the future hospital, making them more specialized and smaller [33] while they use their expanded networks for some kinds of services.

Future hospitals will network with other healthcare providers to share knowledge, experiences, and resources. They will collaborate to identify and respond to community health needs. The network structure allows for economies, proper consumption of resources, development of innovative capabilities of hospitals, reduction of the negative aspect of competition between hospitals, creation of a sustainable market-based health system, and ensuring effective care of patients [12, 34]. The map of the related structural and role elements is presented in Fig. 8. The next aspect of future hospitals is the way they create and use knowledge.

### Knowledge and research

Future hospitals will play a more active role in knowledge production by using different research methods and knowledge translation. These hospitals will provide the context of the activities of researchers and facilitate start-ups. Attention to social medicine and educational issues will be strengthened. Health environmental monitoring units will be established to identify and manage existing and future changes and trends. Due to rapid growth and progress in the health system, the field of "health innovation" will be supported, and it examines and monitors health developments and innovations at the global level to increase confidence and reduce risks [35]. The knowledge and research-related elements of the future changes are presented in Fig. 9 as follows. The third feature of future hospitals is the variation in their service delivery structure.

### Service delivery

Hospitals will continue their service delivery through multidisciplinary teams and teamwork-based. Patient-centered and providing teamwork services will be necessary to meet the needs of the future [36]. Absolute coordination with other providers is another point. Schurr quotes Thomas Bodenheimer "*Patients with multiple chronic diseases are likely to see about 16 doctors a year. In the meantime, treatments and diagnostic processes may be repeated by doctors, which is dangerous and also costly for patients*" [37]. One example of lacking the integrated model of services delivery is patient mortality, about one-third of deaths in the United States are due to acute myocardial infarction in the post-hospital discharge phase [38]. These problems need even more coordination between hospitals and health service providers in the future to ensure the population’s health and prevent and reduce errors. Obviously, the service delivery change needs variation in health forces and also a new generation of forces will emerge.

### Health forces

In the future, hospital staff will come from new generations with different characteristics than those in the past. Generation Z, born after 1995, has grown up in the digital age and is accustomed to using technology. As depicted in Fig. 10, they tend to be self-reliant, value freedom, rely heavily on technology, and prioritize speed. They spend most of their time online and have limited interaction with the real world, which is reinforced by the metaverse. This generation seeks a work-life balance, values friendly relationships in the workplace, desires support for personal development, and looks for opportunities to flourish and prove themselves [39]. To attract and retain them, managers must create appealing work environments that offer factors like flexible working hours and remote work arrangements.

One of the critical issues that the health system workforce will face in the future is the need for interdisciplinary competencies and the ability to work in collaborative teams. In recent years, there has been increasing pressure in higher education to train graduates in health systems who can effectively collaborate across disciplines to solve complex problems. An approach to interprofessional education has been developed for this purpose [40].

In addition, there will be an increased emphasis on improving health literacy among hospital staff. High health literacy among healthcare workers is assumed to lead to improved health literacy among patients. There is a positive correlation between health literacy and the health status of the population, and a negative association with health inequalities [41]. Therefore, hospital and university managers must take steps to improve the health literacy of the future health system workforce. The following section will discuss the political and legal changes that will affect hospitals in the future.

### Political and legal

According to Rechel [28], regulating hospitals and their services is not solely a technical or managerial issue, but also a political decision. Various political and legal trends will impact the future of hospitals. Experts have highlighted that given the location of the Middle East and continuous conflicts and terrorism in the region, planners of future hospitals should always consider the threats caused by these factors. In recent years, political communication and exchanges between countries have grown, expanding the phenomenon of globalization. While globalization can potentially increase access to healthcare, it can also indirectly affect society’s health by increasing competition among companies and impacting working conditions and wages. Moreover, globalization increases the possibility of spreading infectious diseases like COVID-19 but can also improve low-income countries’ health quality [42]. Today, governments try to communicate with each other and use their growth and development potential. Globalization provides an opportunity for developing countries’ health systems to learn from best practices worldwide or attract resources for their growth by establishing active health diplomacy.

### Economical

As presented in Fig. 11, individuals without insurance have a higher mortality rate from cancer [43]. This underscores the importance of ensuring that all individuals have access to affordable and comprehensive health insurance coverage, especially for chronic diseases like cancer. Moreover, society’s demand for insurance coverage for chronic and palliative diseases is expected to increase with the rise in the population’s average age and elderly-related ailments.

Marketing skills will also be a crucial tool for success in healthcare systems, particularly hospitals, in the future. Service research, design, and strategic marketing are vital components of designing and providing effective healthcare services [44]. Consequently, it is essential for managers and decision-makers in the healthcare industry to support and empower their workforce with these skills to gain a competitive edge in the market.

Overall, both insurance coverage and marketing skills play critical roles in shaping the future of healthcare delivery. By investing in these areas, healthcare organizations can improve patient outcomes and stay ahead of the curve in an ever-changing healthcare landscape.

## Population and disease

The risk of emerging and spreading infectious diseases is rapidly increasing due to global environmental changes and increased migration [45]. The COVID-19 pandemic has shown that emerging diseases can quickly become a global issue and impact all aspects of life, including social, economic, and health. Therefore, planning and designing future hospitals based solely on chronic or elderly-related diseases would be too simplistic. The COVID-19 pandemic has taught us the importance of hospital flexibility for potential upcoming challenges.

In addition to considering infectious diseases, it is a fact that the world’s population is aging due to declining fertility rates and increased life expectancy [46]. According to the World Health Organization, approximately 40 million people require palliative care every year, with about 78% residing in low-income and middle-income countries. Shockingly, only about 14% of people who require palliative and end-stage care receive it. Over the past few decades, end-of-life care in hospitals has become increasingly complex [47]. In the future, special attention must be given to hospitals to provide services to chronic patients and palliative care. It will be necessary for hospitals to collaborate with informal providers inside patients’ homes and care centers outside the hospital and support them technically.

Antimicrobial resistance poses another challenge for the future of global health. This challenge has the potential to cost billions of dollars annually to the global economy and endanger the lives of ten million people by 2050 [7]. Hospitals must constantly be reminded of these issues and leverage their scientific potential, equipment, and staff to proactively address future challenges.

### Technology

Technology development is one of the most emphasized future changes for hospitals and is advancing faster than ever. Digital health is considered one of the main drivers of change in the future of healthcare systems [48]. Wearable sensors, mobile health technologies, information technology, telemedicine, and network systems have made it possible to provide health services and remotely monitor health parameters, particularly for chronic patients. This results in effective disease management and improved patient quality of life [49, 50]. The convergence of different branches of technology will change service processes as well as physical design [51]. Comprehensive health systems use intelligent technologies that enable patients to self-manage their health symptoms, improve treatment decisions at the right time, and enhance medication adherence [49].

Thanks to new, more effective and personalized medicine, patients are living longer, and many previously incurable diseases are now well-managed as chronic conditions [37]. While technology has many advantages, it is important to note that it can also significantly increase healthcare costs. Additionally, these technologies do not always lead to improved clinical outcomes, and safety must be considered [52]. In future hospitals, it will be necessary to have a systematic, transparent, and impartial evaluation of health technology. The technological-related elements of the map are presented in Fig. 6.

### Values and philosophy

The concepts, principles, and values of different stakeholders in hospitals have already begun changing and will continue to do so. Patients are no longer passive recipients of services but active participants with increasing expectations for value, satisfaction, and experience [44]. Laurent Gille and colleagues recognize that the determinants of healthcare demand will change, and believe that in the medium and long term, focus will shift from the right to effective treatment to the duty of being healthy [53].

In the future, hospitals will increasingly involve patients in treatment processes. One option being considered is changing the name of hospitals from "Hospital" to "Health Homes." Empowering and improving patients’ living conditions will be one of the missions of hospitals, with the aim of improving health outcomes and reducing the workload of hospitals. Ahn also highlights that empowering people with chronic diseases to manage their conditions independently would improve health while lowering costs [54].

### Environmental

The pollution of air, water, and soil has increased significantly in recent years due to industrial reasons and the use of vehicles. If there is no intervention, this trend is predicted to continue. Most notably, low and middle-income countries and rapidly industrializing nations are expected to experience the most significant increase in pollution [55]. Air pollution also poses a considerable health challenge in developing countries such as Iran [56]. Unfortunately, Iran ranks fifth globally in terms of plastic consumption, with nearly half a million tons of plastic used annually, producing about 5000 tons of waste daily. Pollution caused by plastic particles can have severe health risks [57]. All forms of pollution were responsible for about 9 million premature deaths in 2015, accounting for 16% of all deaths worldwide. Additionally, it is estimated that pollution causes around 268 million disability-adjusted life-years (DALYs) [58].

Climate change is another challenge that has intensified in recent decades, affecting communities’ health through various means. These include climate change’s effect on the spread of infectious diseases, changes in workforce capacity, and food security [59]. Unfortunately, Middle Eastern countries will be more affected by the negative consequences of climate change than other regions [60]. By the year 2100, the average temperature in most parts of Iran is expected to increase by more than 4 degrees Celsius [61]. Therefore, diseases related to pollution and climate change are expected to become a significant concern for future hospitals.

## Conclusions

Many changes in the healthcare system have been ongoing and will continue to do so, with increasing intensity and speed. This study focused on Iran as a developing country and had a time horizon of 15 years. However, wild card events may occur that could significantly impact the trends mentioned in this study. Future studies should identify these wild cards and evaluate their effects on future changes. The changes described in this study have synergies and interactions that can shape different future scenarios. It is recommended to conduct further research in this regard.

Additionally, establishing environmental monitoring units at both the macro level of the health system and within hospitals is suggested to monitor these changes for informed decision-making and planning. The main limitation of this study was the Covid-19 outbreak, which caused difficulties in conducting interviews and holding expert panels. However, the research team utilized virtual communication facilities to overcome these issues.

This study categorized future changes in hospitals using a mind map, which has several advantages. We hope that hospital managers and future leaders of the healthcare system will use a forward-thinking approach when planning for the future. As Erwin noted, hospital managers should ask themselves whether their current management approach is optimal [2].

## Limitations of the study

COVID-19 made the research team use virtual interviewing, which made the interviewing process more complex and time-taking. Due to the objection of some interviewees to audio recording, their interviews were recorded in writing, and it restricted access to some participants, Which required many follow-ups.

## Data Availability

All data generated or analyzed during this study are included in this published article.

## Abbreviations

MOHME: Ministry of Health and Medical Education
UHC: Universal health coverage
HI: Health insurance
HP: Health care Provider
HM: Hospital Management
RFM: Researchers and faculty members
